# Detection of Tick-Borne Pathogen Coinfections and Coexposures to Foot-and-Mouth Disease, Brucellosis, and Q Fever in Selected Wildlife From Kruger National Park, South Africa, and Etosha National Park, Namibia

**DOI:** 10.1155/tbed/2417717

**Published:** 2024-12-12

**Authors:** Carlo Andrea Cossu, Sunday Ochonu Ochai, Milana Troskie, Axel Hartmann, Jacques Godfroid, Lin-Mari de Klerk, Wendy Turner, Pauline Kamath, Ockert Louis van Schalkwyk, Rudi Cassini, Raksha Bhoora, Henriette van Heerden

**Affiliations:** ^1^Department of Veterinary Tropical Diseases, Faculty of Veterinary Science, University of Pretoria, Onderstepoort 0110, South Africa; ^2^Antimicrobial Research Unit, College of Health Sciences, University of KwaZulu-Natal, Durban, South Africa; ^3^International Centre for Antimicrobial Resistance Solutions, Copenhagen S 2300, Denmark; ^4^Etosha National Park, Ministry of Environment, Forestry and Tourism, Etosha Ecological Institute, Okaukuejo, Namibia; ^5^Department of Arctic and Marine Biology, Faculty of Biosciences, Fisheries and Economics, UiT–The Arctic University of Norway, Tromsø, Norway; ^6^Office of the State Veterinarian, Department of Agriculture, Land Reform and Rural Development, Kruger National Park, P.O. Box 12, Skukuza 1350, South Africa; ^7^U.S. Geological Survey, Wisconsin Cooperative Wildlife Research Unit, Department of Forest and Wildlife Ecology, University of Wisconsin-Madison, 1630 Linden Dr., Madison 53706, Wisconsin, USA; ^8^School of Food and Agriculture, University of Maine, Orono 04469, Maine, USA; ^9^Maine Center for Genetics in the Environment, University of Maine, Orono 04469, Maine, USA; ^10^Department of Migration, Max Planck Institute of Animal Behavior, Radolfzell, Germany; ^11^Department of Animal Medicine, Production and Health, University of Padova, Legnaro (PD), Italy

**Keywords:** brucellosis, epidemiology, foot-and-mouth disease, Q fever, tick-borne disease, wildlife disease, zoonosis

## Abstract

**Background:** Although the rate of emerging infectious diseases that originate in wildlife has been increasing globally in recent decades, there is currently a lack of epidemiological data from wild animals.

**Methodology:** We used serology to determine prior exposure to foot-and-mouth disease virus (FMDV), *Brucella* spp., and *Coxiella burnetii* and used genetic testing to detect blood-borne parasitic infections in the genera *Ehrlichia*, *Anaplasma*, *Theileria*, and *Babesia* from wildlife in two national parks, Kruger National Park (KNP), South Africa, and Etosha National Park (ENP), Namibia. Serum and whole blood samples were obtained from free-roaming plains zebra (*Equus quagga*), greater kudu (*Tragelaphus strepsiceros*), impala (*Aepyceros melampus*), and blue wildebeest (*Connochaetes taurinus*). Risk factors (host species, sex, and sampling park) for infection with each pathogen were assessed, as well as the prevalence and distribution of co-occurring infections.

**Results:** In KNP 13/29 (45%; confidence interval [CI]: 26%–64%) kudus tested positive for FMD, but none of these reacted to SAT serotypes. For brucellosis, seropositive results were obtained for 3/29 (10%; CI: 2%–27%) kudu samples. Antibodies against *C. burnetii* were detected in 6/29 (21%; CI: 8%–40%) kudus, 14/21 (67%; CI: 43%–85%) impalas, and 18/39 (46%; CI: 30%–63%) zebras. A total of 28/28 kudus tested positive for *Theileria* spp. (100%; CI: 88%–100%) and 27/28 for *Anaplasma/Ehrlichia* spp. (96%; CI: 82%–100%), whereas 12/19 impalas (63%) and 2/39 zebra (5%) tested positive for *Anaplasma centrale*. In ENP, only 1/29 (3%; CI: 0%–18%) wildebeest samples tested positive for FMD. None of the samples tested positive for brucellosis, while *C. burnetii* antibodies were detected in 26/30 wildebeests (87%; CI: 69%–96%), 16/40 kudus (40%; CI: 25%–57%), and 26/26 plains zebras (100%; CI: 87%–100%). A total of 60% *Anaplasma/Ehrlichia* spp. and 35% *Theileria/Babesia* spp. in kudu and 37% wildebeest tested positive to *Theileria* sp. (sable), 30% to *Babesia occultans*, and 3%–7% to *Anaplasma* spp. The seroprevalence of Q fever was significantly higher in ENP, while *Brucella* spp., *Anaplasma*, *Ehrlichia*, *Theileria*, and *Babesia* species were significantly higher in KNP. Significant coinfections were also identified.

**Conclusion:** This work provided baseline serological and molecular data on 40+ pathogens in four wildlife species from two national parks in southern Africa.

## 1. Introduction

Wildlife are often linked with emerging infectious diseases relevant to human and animal health and are considered to be the source of 70% of zoonoses worldwide (USGS 2024) [1, 2]. Several studies have highlighted the wide range of pathogens that wild animals may carry without necessarily showing overt clinical signs [3–9]. Multiple endemic diseases (i.e., bovine tuberculosis, brucellosis, rabies, Ebola, and leptospirosis) have been associated with a wildlife source, and their management imposes serious challenges at the wildlife/human interface [8–15]. As a result of increased mortality, reduced productivity, costs related to disease control, losses in trade, decreased market value, and food insecurity, wildlife-emerging diseases constitute an additional and important threat to the economy of the livestock industry [16, 17]. Moreover, many wildlife diseases have caused important decreases in endangered animal populations, affecting their conservation status [18]. Most infectious diseases are still largely neglected in wildlife, especially those that are endemically persistent and do not cause obvious clinical signs or have a long incubation period.

In this study, we investigated exposure to foot-and-mouth disease virus (FMDV), *Brucella* spp., and *Coxiella burnetii*, as well as infection with several tick-borne pathogens (TBPs) (*Anaplasma*, *Ehrlichia*, *Theileria*, and *Babesia* spp.) in greater kudu (*Tragelaphus strepsiceros*), plains zebra (*Equus quagga*), impala (*Aepyceros melampus*), and blue wildebeest (*Connochaetes taurinus*) from two national parks, namely, Kruger National Park (KNP), South Africa, and Etosha National Park (ENP), Namibia. FMDV causes foot-and-mouth disease (FMD), a World Organization for Animal Health (WOAH) listed disease that has been reported from more than 70 wildlife species [6, 19]. FMD is endemic in various African countries (e.g., South Africa, Mozambique, and Zimbabwe) and has a negative impact on the national economy of a disease-endemic setting, and also has the potential to spread across boundaries [20]. The circulation of FMDV in wildlife represents a significant burden on wildlife management and conservation of endangered species [21, 22]. In livestock animals, FMD primarily occurs in an acute form with fever, lameness, inappetence, and the formation of vesicles in and around the mouth and on the feet. Clinical signs are often severe in pigs, obvious in cattle, and mild in sheep and goats [23]. Clinical FMD in wildlife seems to be a rare event, but it can occasionally be devastating to some species of antelope as has been documented in South Africa in impala (*A. melampus*) [24] and in mountain gazelles (*Gazella gazella*) in Israel [25].

Important subsets of infectious diseases that are neglected in wildlife include intracellular bacterial pathogens. Inter alia, *Brucella* spp. and *C. burnetii* cause important veterinary and zoonotic diseases worldwide. Brucellosis is a disease of great economic importance, especially for the livestock industry, causing significant production losses and impediments to trade and exportation [26]. Brucellosis has been recorded in a wide range of African wildlife, but the effect of the disease in sylvatic settings has been largely ignored and understudied. The circulation of the pathogen in wildlife raises challenges for disease control and management. For instance, France was bovine brucellosis-free since 2005 but experienced bovine and human cases due to *B. melitensis* in 2012 in the French Alps. The investigation identified spillover from wild Alpine ibex (*Capra ibex*) to domestic ruminants [27]. Few serological tests have been validated for use in wild animal species. The standard indirect enzyme-linked immunosorbent assays (ELISAs) are designed to be specific to livestock species and thus limited for wildlife testing. As none of the serological tests are 100% sensitive and specific [28], the criteria for seropositive brucellosis diagnosis require two positive test results in series. Q fever is an emerging disease caused by bacterium *C. burnetii* which has a high impact on public health, animal health, and economy. It is listed by WOAH as a multispecies disease of concern for its high zoonotic potential, worldwide distribution, airborne spread, persistent infection (potentially lifelong), and direct production losses for the dairy industry (abortions, dead or weak offspring, infertility, and metritis). *Coxiella burnetii* is severely under-reported and underappreciated throughout Africa [29, 30], even though wildlife have been demonstrated to play an important role in Europe and elsewhere [31–33].

Among the emergent threats, TBPs have a great impact on animal and human health throughout the African continent [29, 34]. The epidemiology of ticks and TBPs is complex and multimodal such that environmental variables and contact among wildlife, livestock, and humans influence the transmission dynamics of TBPs. Therefore, wildlife losses and climate changes may result in the increase of disease risk [35]. *Anaplasmataceae* and *Piroplasmida* are two major taxa of obligate intracellular pathogens transmitted by blood-sucking arthropods (especially ticks). Members of the family *Anaplasmataceae* are frequently reported in African wildlife, especially African buffalo (*Syncerus caffer*) and several antelope species [36–39]. The most important tick-borne diseases affecting livestock in Africa are *Theileria parva* (East Coast fever, January disease, and corridor disease), *Ehrlichia ruminantium* (heartwater), *Anaplasma marginale* (gallsickness), *Theileria annulata* (tropical theileriosis), *Babesia bovis*, and *Babesia bigemina* (Asiatic and African redwater, respectively) [40]. Anaplasmosis, heartwater, theileriosis, and babesiosis are known to cause 18% of reported cattle mortalities in South Africa [41].

KNP in South Africa is classified as an endemic zone for FMD and an infected zone for brucellosis and corridor disease, where sporadic outbreaks are reported [19, 42]. In contrast, ENP is a protected, noninfected FMD zone with no brucellosis detected in wildlife. According to the systematic review performed by Simpson et al. [7], three prevalence studies have been conducted on *Brucella* spp. in Namibian wildlife, all of them reporting negative results although based on small sample sizes, that is, 0/23 white rhinoceros (*Ceratotherium simum*) and 0/9 black rhinoceros (*Diceros bicornis*) from Waterberg National Park [43], 0/27 impala from ENP [44], and 0/122 farmed springbok (*Antidorcas marsupialis*) and gemsbok (*Oryx gazella*) [45]. Only one publication investigated and reported the presence of *C. burnetii* in KNP wildlife, that is, in vervet monkeys (*Chlorocebus pygerythrus*) [46] with no investigations or reports on *C. burnetii* available from ENP, highlighting the lack of research on these diseases in South African wildlife. The two parks differ in many aspects, with the presence of African buffalo in KNP being the main difference that might play a significant role in disease dynamics. The objectives of this study were to (1) assess the presence/absence and estimate the infection sero/prevalence of selected pathogens in four free-ranging wild animal species in KNP and ENP; (2) evaluate risk factors for infection, including animal species, sex, and sampling park; and (3) assess significance of coinfections and/or coexposure to multiple pathogens.

## 2. Materials and Methods

### 2.1. Study Design

**Figure 1:**
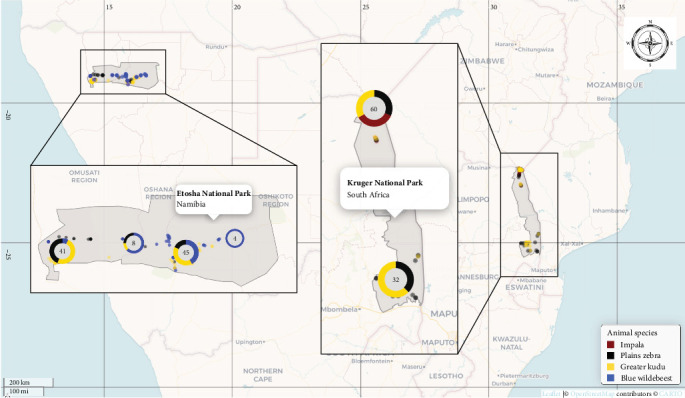
Spatial distribution of serum and EDTA blood samples collected in Kruger National Park (right) and Etosha National Park (left). Color legend stratifies samples per animal species, including impala (*Aepyceros melampus*), plains zebra (*Equus quagga*), greater kudu (*Tragelaphus strepsiceros*), and blue wildebeest (*Connochaetes taurinus*).

Whole blood and serum samples were collected during May 2018 to September 2019 from two national parks, KNP and ENP, and the host species targeted in this study included free roaming greater kudu (*n* = 72; 32 from KNP and 40 from ENP), plains zebra (*n* = 65; 39 from KNP and 26 from ENP), impala (*n* = 21 from KNP), and blue wildebeest (*n* = 30 from ENP) (Figure 1). These samples were originally tested for the presence of antibodies against *Bacillus anthracis* (causal agent of anthrax) [47]. The sample size was small due to budget constraints as the animals were chemically immobilized and collared to monitor their movement and exposure to *B. anthracis* in KNP and ENP [48]. In the framework of the present work, the same samples were also screened using serology to detect FMDV, *Brucella* spp. and *C. burnetii*, and DNA from blood were analyzed using a molecular reverse line blot (RLB) method to detect *Anaplasma*, *Ehrlichia*, *Theileria*, and *Babesia* spp.

Each animal was selected randomly from different herds. All animals were adults or subadults as was required for the collaring study. Each sample was assigned a unique identification number. Supporting information data on sampling date and GPS location were recorded.

### 2.2. Study Area

KNP is situated in the Limpopo and Mpumalanga provinces of South Africa. It is regarded as one of the largest and most important national parks in Africa, hosting a total of 148 wild mammal species, including the big five (i.e., lion, leopard, elephant, rhino, and buffalo), in a 19,485 km^2^ fenced conservation area situated in the FMD infected zone [49]. Population estimates for the selected wildlife species in KNP include 11,200–17,300 greater kudus, 132,300–176,400 impalas, and 23,700–35,300 plains zebras (https://www.sanparks.org).

ENP, also situated in the FMD protected zone [50], is an almost 23,000 km^2^ wildlife reserve located in northern Namibia. ENP is home to 114 mammal species, but it is not considered a big five reserve as African buffaloes are not present in the park [51]. Aerial estimates of selected wildlife include 2822–5592 blue wildebeests, 11,338–17,126 plains zebras [51], and 394–580 greater kudus [52].

### 2.3. Laboratory Protocols

#### 2.3.1. Blood-Borne Parasite Detection

The PureLink DNA extraction kit (Invitrogen, Germany) was used to extract DNA from 200 μL of each blood sample according to the manufacturer's instructions and eluted in 100 μL of elution buffer. The RLB hybridization assay was performed as previously described [53–56] to detect *Theileria*, *Babesia*, *Ehrlichia*, and *Anaplasma* species. Negative and known positive controls were included for each pathogen species. The probes included in the RLB membrane are listed in Supporting Information 1: Table S1.

#### 2.3.2. Serological Tests

For serological screening, we employed commercially available ELISA kits produced by ID-VET. The ID Screen FMD is a nonstructural protein competitive ELISA (NSPCE) and was used for the detection of antibodies against the 3ABC proteins of FMDV. Similarly, the ID Screen Brucellosis Serum Indirect Multi-species ELISA was used to detect antibodies against the lipopolysaccharide (LPS) of smooth *Brucella* spp., while the ID Screen Q Fever Indirect Multi-species ELISA was used in the detection of antibodies against *C. burnetii* antigenic phases I and II. All the serum samples were run in duplicates, and the coefficient of variation (%CV) was ensured to be less than 20% for all duplicates and less than 10% overall. FMDV SAT serotyping of NSPCE-positive sera were tested by Agricultural Research Council–Onderstepoort Veterinary Institute (ARC-OVI), South Africa, and Central Veterinary Laboratory in Namibia for all serotypes with a solid-phase cELISA (SPCE). SPCE is the official screening test in South Africa and Namibia.

For *Brucella* spp., serum was first screened using rose Bengal test (RBT) obtained from Onderstepoort Biological Products (OBP) as per the manufacturer's instruction with the *Brucella*-positive serum from OBP. Sera were analyzed using ID-VET Multi-species indirect ELISA (iELISA) as per the manufacturer's instructions. Negative RBT sera were tested with iELISA in pools of 10 animals grouped by species. If positive reactions were obtained in the pools, the samples were re-tested individually. Animals were confirmed seropositive only if positive to both RBT and iELISA due to the well documented problem of extensive serological cross-reactions with other bacteria [57].

### 2.4. Data Analysis and Reporting

Data were analyzed in R programming language (version 4.2.1) using the R studio IDE software (RStudio Team, 2021). To account for our small sample sizes, confidence intervals and hypothesis testing were estimated employing exact/nonparametric methods, and the results were interpreted with great caution. The 95% confidence intervals (CIs) were calculated to measure variability and error of our estimated point prevalences by species. Because of small sample sizes, we opted for the more conservative Clopper–Pearson method [58] using the R function “exactci” from the “PropCIs” package.

To determine which infections were most likely to co-occur in hosts, we used the Spearman's correlation coefficient (*r*_s_) using function “cor” (with method = “Spearman”) from package “stats” in R. Coefficient (*r*_s_) values from 0 to 0.25 or from 0 to −0.25 indicate the absence of correlation, whereas values from 0.25 to 0.50 or from −0.25 to −0.50 point to poor correlation between variables; values ranging from 0.50 to 0.75 or −0.50 to −0.75 are regarded as moderate to good correlation, and *r* values from 0.75 to 1 or from −0.75 to −1 indicate very good to excellent correlation between variables [59]. This correlation was considered significant if the *t* test for Spearman rank correlation indicated a *p*-value <0.05 under the null hypothesis of no correlation [58]. When performing multiple comparisons, the family-wise error rate increases, hence the probability of finding at least one false positive (type I error) [60]. To yield conservative results, *p*-values were adjusted using the Bonferroni correction in which the *p*-values are multiplied by the number of comparisons [61]. This was achieved by applying function “p.adjust” (method “bonferroni”) from package “stats.”

To assess correlation between the prevalence and independent variables (i.e., animal species, sex, and sampling park), we employed the chi-squared test. An alternative when the conditions for a chi-squared test are not met (i.e., no cells with expected values <1, and no more than 20% of cells with values <5), is a Monte Carlo simulation [62] performed with the option “simulate.p.value = TRUE” in the function “chisq.test.” We set the number of replicates in the simulation to *B* = 2000. Again, *p*-values were adjusted using the Bonferroni correction, and statistical level was set at *α* = 0.05.

## 3. Results

A summary of the laboratory diagnostic results, including estimates and errors (95% CIs) of prevalences in each animal species and park are reported in Table 1.

The NSP-cELISA for FMDV detected antibody in the sera of 13 greater kudu samples (40.6%; 13/32) from KNP, 12 of which had high titers (i.e., 10 <SN <30; Supporting Information 2: Figure S1; Table 1). These animals were sampled during October 2018, mostly in the northern area of KNP. Only four of the 12 FMDV-positive kudu samples were tested with SPCE ELISA due to financial constraints, none of which could be serotyped and thus interpreted as negative by SPCE. In ENP, only one blue wildebeest (3.3%; 1/30), sampled near Ozonjuitji m'Bbari (Central ENP) in July 2018, tested weakly positive using the NSP-cELISA for FMDV but tested negative using SPCE.

For *Brucella* spp., the first serological screening with RBT indicated four clear positive sera (three kudus and one zebra from KNP), and an additional five (two kudus and one zebra from KNP and two wildebeest from ENP) were regarded as suspect due to faint positive reaction. At the second testing with the commercial *Brucella* spp. iELISA, seven animals tested positive and one suspect. From KNP, 3/29 kudus (10%) tested positive using both serological techniques and were thus considered seropositive. Additionally, eight greater kudus (28%; 8/29), one impala (5%; 1/21), and three plains zebras (9%; 3/35) tested positive using either the RBT or iELISA assay and were regarded as negative results. The brucellosis-positive animals originate from KNP and were sampled mostly in the northern part of KNP. No animals in ENP were positive for *Brucella* spp.

A summary of *C. burnetii* serology is reported in Table 1. As a general trend, the prevalence of antibodies against *C. burnetii* in all samples collected from ENP (71%) was much higher than those collected from KNP (43%). We also report the presence of several strong reactions, that is, high iELISA titers in most individuals (Supporting Information 2: Figure S1).

**Figure 2:**
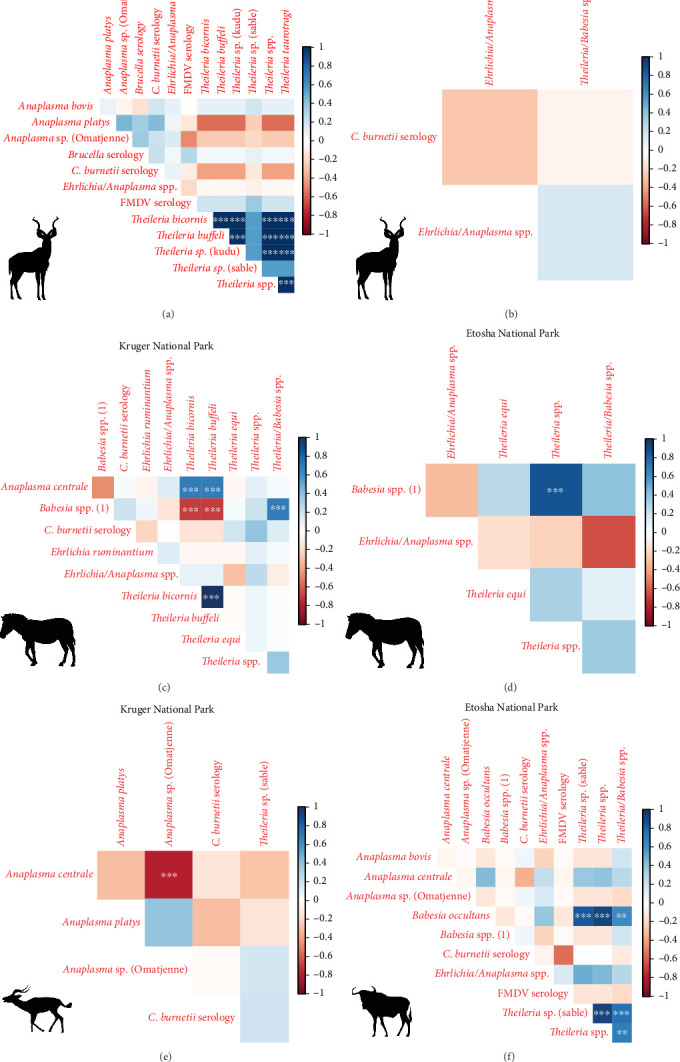
Correlation matrix representing correlation coefficients for concurrence of pathogen infection/exposure in kudu (*Tragelaphus strepsiceros*) (A, B), zebra (*Equus quagga*) (C, D), impala (*Antidorcas marsupialis*) (E), and wildebeest (*Connochaetes taurinus*) (F) from Kruger National Park (left panel) and Etosha National Park (right panel). Blue squares indicate positive correlation, and red squares indicate negative correlation. Color intensity indicates strength of correlation. Asterisks indicate significant correlation: *⁣*^*∗∗∗*^ = *p*-value < 0.001; *⁣*^*∗∗*^ = *p*-value < 0.01; *⁣*^*∗*^ = *p*-value < 0.05. *p*-values were adjusted with Bonferroni correction.

We investigated coinfection and coexposure to the different pathogens (Figure 2). We highlight that in kudu from KNP, *T. buffeli*, *T. bicornis*, *Theileria* sp. (sable), and *Theileria* sp. (kudu) occurred almost always together. In zebra from KNP, *T. bicornis* and *T. buffeli* occurred always together and were positively correlated with *A. centrale* (*p* < 0.001; *r*_s_ = 0.7) but negatively correlated to *Babesia* spp. (*p* < 0.001; *r*_s_ = −0.7). On the other hand, in zebra from ENP positivity to the *Theileria* spp. probe was positively correlated to the *Babesia* spp. (1) probe (*p* < 0.001; *r*_s_ = 0.87). In impala from KNP, infection with *A. centrale* was negatively correlated to infection with *Anaplasma* sp. (Omatjenne) (*p* < 0.001; *r*_s_ = −0.78). In wildebeest from ENP, *B. occultans*-infected animals were almost always coinfected with *Theileria* sp. (sable). Interestingly, one kudu from KNP (ID: TS-E-10, female, adult, sampled in KNP) bore most infections/exposures at the same time, as it was seropositive to FMDV, *Brucella* spp., and *C. burnetii*, and coinfected with *A. platys*, *Anaplasma* sp. (Omatjenne), *T. bicornis*, *T. buffeli*, *Theileria* sp. (kudu), *Theileria* sp. (sable), and *T. taurotragi*. According to the Pearson's chi-squared test (with Monte Carlo replicates), the variables “sampling park” and “animal species” were the most associated with pathogen prevalence and seroprevalence (Table 2).

## 4. Discussion

This study established baseline data of infection with tick-borne diseases as well as exposure to FMD, Q fever, and brucellosis in four wild animal species in two national parks. Laboratory analysis revealed very high prevalence (70%–100%) of *Theileria/Babesia* and *Anaplasma/Ehrlichia* spp. infection in kudu, impala, and zebra from KNP. Moreover, most or even all of the zebra and wildebeest sampled in ENP were seropositive for Q fever. Indeed, the seroprevalence of Q fever was found to be significantly higher in ENP, while *Brucella* spp., *Anaplasma*, *Ehrlichia*, *Theileria*, and *Babesia* species were significantly higher in KNP.

### 4.1. *Anaplasma/Ehrlichia* and *Theileria/Babesia* Prevalences are Higher in KNP Compared to ENP

As highlighted by the comparison of the 95% CI and the chi-squared statistics, infection prevalences of *Anaplasma/Ehrlichia* and *Theileria/Babesia* genera were significantly higher in KNP compared to ENP in both kudu and zebra. This may be due to the relative diversity and abundance of ticks inhabiting the parks. Indeed, the prevalence of tick infestation in ENP wildlife is reportedly well below those reported in other parts of southern Africa [63–65]. Tick distribution and ultimately the survival of pathogens in ticks and animal hosts are, in turn, affected by abiotic factors. Indeed, hot dry conditions and desiccating winds adversely affect the population of questing ticks by imposing mortality on unfed ticks [66]. Moisture-related indices significantly affect the presence of ticks and TBDs, with wetter conditions almost always beneficial [66]. ENP is located in a semiarid region of Namibia characterized by a large salt pan, which may be dry for extended periods of the year, especially during the dry season [67]. On the other hand, KNP is situated in northeastern South Africa and has a more diverse climate with a greater availability of water throughout the year compared to ENP. Overall, ENP is considerably drier than KNP and therefore a less suitable region than KNP for tick proliferation, infestation and transmission of TBDs. For instance, *Amblyomma hebraeum*, *Amblyomma variegatum* (vectors of *E. ruminantium*), *Rhipicephalus decoloratus* (vector of *Babesia bigemina* and *A. marginale*), and *Rhipicephalus appendiculatus* (vector of *Theileria parva* and *A. bovis*) are present mainly or only in KNP, whereas *Hyalomma rufipes* (vector of *Babesia occultans*), *Hyalomma truncatum* (vector of several *Anaplasma/Ehrlichia* spp.), and *Rhipicephalus evertsi* (vector of *T. equi* and *B.caballi*) are found in both parks [68–70].

### 4.2. High Prevalence and Coinfection of *Theileria* spp. in Kudu and Impala From KNP

In the present study, we report extremely high prevalence of *T. buffeli* and *T. bicornis* in 27/28 kudus (96%; CI: 82%–100%) and 19/19 impalas (100%; CI: 82%–100%) from KNP. In addition, in KNP kudu, there was high prevalence (90%–100%) and significantly associated coinfections of pathogens from the genera *Theileria*, including *T. taurotragi*, *T. buffeli*, *Theileria* sp. (kudu), and *Theileria* sp. (sable) (Table 1). *Theileria* spp. (sable) was also detected in 5/19 impalas (26%; CI: 9%–51%) from KNP. None of the 40 kudus from ENP tested positive for any of the tested *Theileria* species.

*Theileria taurotragi* and *T. buffeli* are “schizont non transforming” *Theileria* spp. and therefore classified as benign parasites, with rare clinical signs that mainly occur due to piroplasm-induced acute hemolytic anemia [71]. Indeed, *T. taurotragi* caused bovine cerebral theileriosis in young African shorthorn cattle [71] and theileriosis in eland (*Tragelaphus oryx*) [71]. *Theileria* sp. (sable) and *Theileria* sp. (kudu) [56] are regarded as pathogenic species in African wild artiodactyls. Mortalities in roan antelope (*Hippotragus equinus*) due to *Theileria* sp. (Sable) have been reported after translocation [56]. Infection with *Theileria* sp. (sable) negatively affects attempts to establish breeding herds and reintroduction efforts into the wild due to calf mortalities [72]. *Theileria bicornis* has not been found to cause mortality but has been reported in free-ranging white and black rhinoceroses in South Africa and Kenya [55, 73, 74], as well as from apparently healthy nyalas (*Tragelaphus angasii*) [75], impalas, eland (*Taurotragus oryx*), and sable antelope (*Hippotragus niger*) in South Africa [76]. The very high *T. bicornis* prevalences obtained in this study in kudu and impala from KNP (Table 1) might raise concerns for the rhino populations as they are already suffering from poaching and stress induced by unavoidable translocations [77, 78].

Further studies may assist in determining the health effects of the above-mentioned *Theileria* infections in wildlife species. Coinfections may alter virulence of pathogens and subsequent disease outcomes in the hosts [79–81]. As a general rule, coinfections may lead to worse health outcomes for hosts and increase within host pathogen titers, altering transmission ecologies. Nevertheless, the impact on animal fitness due to coinfections between pathogenic and benign *Theileria* species appears to be intricate. For instance, apathogenic *T. mutans* and *T. velifera* seem to protect cattle from the detrimental consequences of *T. parva* infection [82]. This could also be our case, with the benign *T. taurotragi*, *T. bicornis*, and *T. buffeli* protecting wild antelopes from the adverse effects of pathogenic *Theileria* sp. (sable) and *Theileria* sp. (kudu), but this hypothesis needs further investigation. The occurrence and effects of coinfection of multiple pathogen species within wildlife populations remains largely unknown. Indeed, understanding dynamics of coinfection or coexposure to different pathogens is useful in improving our knowledge of pathogen epidemiology in wildlife and in the development of risk models for diseases in various epidemiological contexts.

### 4.3. *Anaplasma centrale* in Impala and Zebra From KNP and Wildebeest From ENP

*Anaplasma centrale* and *A. marginale* are closely related species that cause bovine anaplasmosis in cattle [83]. *A. centrale* is known to be less pathogenic than *A. marginale* in domestic animals as it induces a low degree of anemia, with rare clinical outbreaks [84], but it confers immunity against infection by *A. marginale*. Nonetheless, a clinical case of bovine anaplasmosis caused by *A. centrale* was reported in Europe in 2008 [85]. *A. centrale* seems to be largely subclinical in wildlife [38] where it occurs with moderate prevalences (10%–30%), especially in African buffalo, impala, eland, waterbuck (*Kobus ellipsiprymnus*), and blue and black wildebeest (*Connochaetes gnou*) [37–39, 76, 86]. These wild animal species may be able to maintain *A. centrale* much more efficiently than tick vectors. In fact, although experimental transmission of *A. centrale* by ticks (e.g., *Rhipicephalus simus* and *Dermacentor andersoni*) has been proven [87, 88], secretion of this pathogen into tick saliva occurs at a much lower rate than *A. marginale*, and hence, transmission is achieved only when tick numbers are dramatically increased to compensate for the low pathogen load [88]. In addition, *A. centrale* prevalence in ticks is very low in all tick species considered [89], making them an inefficient reservoir for *A. centrale*. In support of this hypothesis, we report infection with *A. centrale* in 12 impalas (63%; 12/19) and two zebra (5%; 2/39) from KNP and in two wildebeest (7%; 2/30) from ENP. The occurrence of *A. centrale* in impala from KNP is not surprising as the pathogen was already reported in the same species and in buffalo, black wildebeest, common eland, and waterbuck from South Africa [37–39, 76, 86], while the occurrence of *A. centrale* in zebra from KNP and wildebeest from ENP is a new finding that sheds light on the geographic and host range of the pathogen.

### 4.4. *Anaplasma platys* in Kudu and Impala From KNP

*Anaplasma platys* is the etiologic agent of thrombocytic anaplasmosis in dogs and is the only recognized *Rickettsiales* species known to infect platelets [90]. After the first description, *A. platys* has been reported worldwide, including the Americas, Eurasia, Africa, and Australia, mainly in tropical and subtropical areas [91–93]. For a long time, *A. platys* was considered only a canine pathogen, but a wider host tropism for *A. platys* has been demonstrated in recent decades. Cases of *A. platys* infection have been reported in cats, goats, cattle, Bactrian camels (*Camelus bactrianus*), red deer (*Cervus elaphus*), sika deer (*Cervus nippon*), and sable antelope [94–101]. Occurrences in atypical hosts have been attributed to *A. platys*-like bacteria [102, 103]. However, *A. platys*-like species cannot be distinguished from *A. platys* based on 16S rRNA as they are very closely related. These *A. platys*-like species in atypical hosts are considered the probable cause of human infections [104], with clinical signs varying from chronic and nonspecific, including headaches and muscle pains [105] to migraines and seizures due to mixed *A. platys*, *Bartonella henselae*, and “*Candidatus Mycoplasma haematoparvum*” infection [106].

*Rhipicephalus sanguineus* is considered the primary vector for *A. platys* [98, 107, 108] which rarely infests impala and kudu. The agent has also been detected in *Haemaphysalis longicornis* and *Ixodes persulcatus* in Korea, *Rhipicephalus turanicus* in Israel, and *Rhipicephalus* spp. in China [98, 109–111].

Here, we found three kudus (11%; 3/28) and one impala (5%; 1/19) positive to *A. platys* by means of RLB hybridization. Given the limited information available on *A. platys* infections in Africa, it is of particular interest to understand the sylvatic cycle of *A. platys* in kudu and impala and which tick vector (if any) is involved in pathogen transmission.

### 4.5. *Babesia occultans* in Wildebeest From ENP

*Babesia occultans* is considered less pathogenic than other *Babesia* species [112]. Observable clinical signs due to infection with *B. occultans* in cows include anorexia, weakness, fever (≤40°C), anemia, and pale mucous membranes. However, unlike *B. bigemina*, *B. bovis*, and *B. divergens* infections, no jaundice, hemoglobinuria, gastrointestinal disorders, and nervous symptoms have been found in cows infected with *B. occultans* [113, 114]. In this study, we identified nine *B. occultans*-positive wildebeest (30%; 9/30). Since its clinical signs are nearly identical to those of piroplasm infections, it is important for local animal health officers and veterinarians to acknowledge the presence of the pathogen and consider it in diagnoses and treatment strategies.

### 4.6. *Ehrlichia ruminantium* in KNP Zebra

Reports of *E. ruminantium* in African nonruminant wildlife are rare and controversial. For instance, *E. ruminantium*-like colonies were detected in brain endothelial cells of a Nigerian African elephant (*Loxodonta africana*) that reportedly died of anthrax [115]. This report requires verification due to the unusual nature of the case and the possible presence of pathogens similar to *E. ruminantium*. Black and white rhinoceroses from Zimbabwe tested serologically positive to *E. ruminantium* using a MAP1 competitive ELISA [116]. However, this technique is known to cross-react with other *Anaplasmataceae* [117], and therefore, no confirmation can be drawn from these findings.

In our study, two plains zebra from KNP tested positive to *E. ruminantium* with RLB. The occurrence of the pathogen in a wild equid could be most likely incidental, but it may still be of epidemiological importance to understand the source of infection and transmission dynamics, for which further molecular characterization of the pathogen may provide significant insights.

### 4.7. Seropositivity to FMDV in Greater Kudu in KNP

A total of 13 greater kudus (41%; 13/32) from KNP sampled in October 2018, South Africa, were found seropositive to FMD by means of NSPCE. While natural infection with FMD has already been reported in greater kudu from Botswana by means of reverse-transcriptase PCR [118, 119], the present study represents the first report of FMD based on NSPCE in greater kudu in South Africa using serology. This test has not been validated for wildlife. Risk factor analysis (Table 2) indicates that greater kudu has significantly higher prevalence of FMD among the affected animal species investigated. The location (sampling park) was a significant predictor of infection. Antibodies against 3ABC complex of FMDV can be detected in a window of between 1 week to 6 months after exposure to the pathogen [120]. These observations point to circulation of FMD in kudu population from the northern area of KNP that were exposed to the pathogen anytime during April to October 2018. Interestingly, this event might have occurred in proximity and just a few months before the January 2019 outbreak in Vhembe district, Limpopo, South Africa in cattle. Greater kudu has been reported to shed the virus up to 160 days after experimental infection, more than any other African non-buffalo bovid (“antelope”), and clinical signs have been reported from this species without mortality [118, 119, 121]. Nonetheless, the role of kudu in maintaining and spreading FMDV is still to be investigated and clarified. This report underscores the importance of further investigation into the role of kudu in the epidemiology of FMD in KNP and validation of FMD serological tests for wildlife. The lack of seropositive kudu from ENP—where buffalo populations are absent—may indicate that the source of infection for kudu in KNP was most likely the contact with FMD-infected buffaloes. As highlighted by Thomson, Vosloo, and Bastos [19] and Hargreaves et al. [122], antelope species (like kudu and impala) infected through contact with buffalo herds within the park have the potential to jump over the fences and transmit the virus to the cattle living in adjacent communal farms. SPCE is the official screening test in South Africa and Namibia for livestock, which is not validated for wildlife. In this study, the SPCE for SAT-1, SAT-2, and SAT-3 was negative in KNP and all serotypes in ENP. However, to our knowledge, this work represents the first attempt of FMD SAT serotyping in African non-buffalo species by SPCE [6]; hence, the sensitivity of the technique in these animals is not known as there has been no report, to our knowledge of SPCE for SAT in kudu and wildebeest. SPCE is serotype-specific meaning that it targets the structural proteins whose aminoacidic variability is per definition the highest among all viral proteins [119]. Antigenic variation is considered more common in wild animal populations, due to repeated exposure and immune selective pressure of a highly diverse population of infected host species [120, 121]. The strains of the serotypes (SAT-1, SAT-2, and SAT-3) coated to the plate of the SPCE may be significantly different than the ones circulating in KNP wildlife, as the SPCE is validated for livestock animals. Hence, the sensitivity of the SPCE might be mildly to markedly lower than the NSPCE, which on the other hand targets a highly conserved component of the FMDV capsid, that is, the 3ABC complex. Alternatively, positive reactions in kudu by NSPCE might be considered as false-positive results, although this is very unlikely due to the high specificity of the test (>99%) which does not depend on a species-specific conjugate (being a competitive ELISA) and also due to the high titers observed in 12 kudus from KNP (38%; 12/32). Additional research and characterization (using VNT or other tests) are strongly expected to shed light on this phenomenon and could be investigated in the future using available samples.

### 4.8. Seropositivity to FMDV in a Blue Wildebeest From ENP

One blue wildebeest (CT05, male, adult; 3%; 1/30) from ENP, Namibia, was found seropositive for FMD by means of NSPCE but seronegative using SPCE. This finding has to be interpreted cautiously because: the positive sample had a S/N percentage close to the ELISA cutoff (Supporting Information: Figure S1); all the other animals (kudu and wildebeest) from the same park, area and sampling period, tested negative by the assay; buffalo, considered the main maintenance host for FMD in wildlife, is not present in ENP [67]. FMD infection in blue wildebeest from Tanzania, Botswana, and Kenya has been reported by means of RT-PCR with serotypes O and A, SAT-1, and SAT-2 [121, 123]. Blue wildebeest may also suffer the clinical disease, developing oral and foot lesions associated with lameness, fever, and inappetence [123]. However, the NSPCE results were not confirmed with SPCE and thus require further investigation using a larger samples size and alternative techniques such as RT-PCR on oropharyngeal lymph nodes.

### 4.9. Confirmed *Brucella* Exposure in KNP Kudu, Questionable for Plains Zebra, Blue Wildebeest, and Impala

Three kudus (10%; 3/29) in KNP could be considered seropositive for *Brucella* spp. These animals reacted to two serological tests, and additional five kudus were positive to only one serological technique. Numerous studies conducted in southern Africa could not find any serological response in greater kudu, although sample sizes were often small (<30) and used serological tests validated for livestock [124–128]. In this study, seropositivity means that kudu were exposed to *Brucella* spp. and it remains unknown whether they are incidental hosts or part of the maintenance host community for *Brucella* spp. in wildlife. Three plains zebra from KNP (9%; 3/35) tested positive either with RBT (two animals) or iELISA (one animal) and were regarded as suspect cases. This is an area for additional research as agglutination reaction to *Brucella* spp. in zebra has been reported by a previous study [129]. The domestic horse, which is evolutionarily related to zebra, has been demonstrated to harbor different *Brucella* spp. (i.e., *B. abortus* and *B. suis* under natural circumstances and *B. canis* after experimental challenge) and may eventually experience clinical signs (fistulous withers, abortion, and other reproductive problems) [130]. Moreover, a study from Nigeria conducted by Bertu et al. [131] isolated *B. abortus* from asymptomatic horses living in a multispecies farm in Nigeria. However, the risk of transmission of brucellosis from equids is still to be clarified as horses have been indicated as dead-end host [132].

### 4.10. Widespread Exposure to *Coxiella burnetii* in KNP and ENP

In this study, a remarkably high number of individuals (57%; 106/185) across all evaluated wild animal species (44/65 zebras, 22/69 kudus, 14/21 impalas, 26/30 wildebeests) tested positive to the *C. burnetii* iELISA (Table 1). We also obtained many strong positive reactions (19%; 35/185) in any species considered (33/65 zebras, 9/69 kudus, 9/21 impalas, 18/30 blue wildebeests). Finally, our seroprevalence estimates were significantly different than those reported by Gakuya et al. [133], where similar wildlife species were investigated in Kenya using the same serological technique (iELISA). These findings led us to assume that *C. burnetii* is ubiquitous in both KNP and ENP and might have a predilection for southern Africa's ecosystems and/or soils. A significantly higher seroprevalence was registered in animals from ENP. Q fever seroprevalence was especially higher in blue wildebeest, plains zebra, and impala. However, the multispecies *C. burnetii* iELISA has only been validated for use in domestic animals and not wildlife and has not been validated for wildlife species as iELISA tests are designed to be host-specific. The use of inaccurate tests could overestimate the prevalence of disease. In multiple species iELISA assays, IgG-binding proteins (such as protein A, protein G, and protein A/G) are suggested and used as conjugates [134–137], but it is not known how these react with every wildlife host species. According to Kelly et al. [135] and Stöbel, Schönberg, and Staak [137], impala, wildebeest, greater kudu, and zebra react weakly with protein A and strongly with protein A/G, while binding affinity with protein G varies; for impala and wildebeest, reactivity is weak, whereas for kudu, it is moderate and for zebra is strong. The binding affinity with protein A/G is particularly strong for kudu [135]. The Q fever iELISA kit employed in this study used protein A/G. Considering all the facts discussed above, additional investigation may determine if kudu is less affected/exposed to *C. burnetii* than the other species.

Further testing on tissues of wild animals matched with investigation in feeding ticks may provide important details for the clarification of Q fever epidemiology in African wildlife. Also, the expansion of *C. burnetii* investigations in predator animals may provide further information on the sylvatic cycle of the pathogen.

### 4.11. Limitations of the Study and Suggestions

We could detect reactions to nonspecific probes for *Anaplasma/Ehrlichia* and *Theileria*/*Babesia* in ENP but not too many of the species-specific probes investigated. This suggests that the strains present in ENP may not be detectable by the probes which were designed for strains occurring in South Africa due to the presence of local SNPs that do not allow binding with RLB probes. Sequencing data could characterize *Anaplasma/Ehrlichia* and *Theileria*/*Babesia* species occurring in ENP wildlife and thus design probes that can hybridize reliably also with these strains. It may also indicate the occurrence of new species that are not reported in the literature.

RLB probes cross-reactions are not infrequent, and a subset of positive samples should be sequenced to confirm specificity of the RLB probes. However, due to funding constraints, we could not sequence nor characterize any positive RLB occurrences. As a future study, it would be particularly interesting to sequence and confirm the occurrence of *A. platys* in kudu and impala and *E. ruminantium* in zebra from KNP, given their relevance for human and animal health. For serology, there is lack of known positive reference material from wild animals. Multispecies ELISA make use of conjugates that react with multispecies with cutoffs that are not animal species-specific. It is ideal to develop and validate ELISA assays specifically tailored for detecting FMDV, brucellosis, and Q fever across a range of wildlife species.

Our prevalence estimates have wide CIs due to small sample sizes and need to be interpreted cautiously. Interpretations and interventions are conducted by considering both the point estimate/prevalence as well as the entire confidence interval; that is where the true population lies with 95% confidence.

Samples used in this study were part of another project that aimed to unravel differences in exposure to anthrax in endemic and nonendemic locations. Although randomization was introduced as much as possible when selecting sampling units, a moderate–high selection bias has to be considered as it is not possible to extract a proper random sample from wildlife. Moreover, due to prior use in other research, the total number of available samples was reduced leading to a slight discrepancy in the number of animals tested for certain pathogens. For instance, out of the total 32 kudu samples collected from KNP, we had only 28 sera and 29 DNA samples available for testing. This depletion meant that for four of the 32 kudus, we had only one of the two sample types available (either DNA or sera but not both).

## 5. Conclusion

With the present study, we report infections and exposure to several pathogens in wild animal species. We provided evidence-based information that increased the knowledge of pathogen/disease epidemiology in natural settings. This work constitutes a baseline of data useful for implementation and improvement of surveillance and monitoring tools, which are highly valuable for public and animal health stakeholders (i.e., farmers, communities, and governments) and lay the foundations for considerable research advancement.

## Figures and Tables

**Table 1:** Seroprevalence of foot-and-mouth disease virus (FMDV), *Brucella* spp., and *Coxiella burnetii* and prevalence of infection of *Anaplasma*, *Ehrlichia*, *Theileria*, and *Babesia* species in blue wildebeest (*Connochaetes taurinus*), kudu (*Tragelaphus strepsiceros*), impala (*Antidorcas marsupialis*), and zebra (*Equus quagga*) from Kruger National Park, South Africa, and Etosha National Park, Namibia.

Pathogen species (dIagnostic)	Positive/tested = prevalence (95% confidence interval)					
	Blue wildebeest	Greater kudu		Impala	Plains zebra	
	Etosha National Park	Etosha National Park	Kruger National Park	Kruger National Park	Etosha National Park	Kruger National Park
*Anaplasma/Ehrlichia* spp. (RLB)	18/30 = **60%** (41%–77%)	24/40 = **60%** (43%–75%)	27/28 = **96%** (82%–100%)	19/19 = **100%** (82%–100%)	4/17 = **24%** (7%–50%)	28/39 = **72%** (55%–85%)
*Anaplasma bovis* (RLB)	1/30 = **3%** (0%–17%)	0/40 = **0%** (0%–9%)	6/28 = **21%** (8%–41%)	0/19 = **0%** (0%–18%)	0/17 = **0%** (0%–20%)	0/39 = **0%** (0%–9%)
*Anaplasma centrale* (RLB)	2/30 = **7%** (1%–22%)	0/40 = **0%** (0%–9%)	0/28 = **0%** (0%–12%)	12/19 = **63%** (38%–84%)	0/17 = **0%** (0%–20%)	2/39 = **5%** (1%–17%)
*Anaplasma platys* (RLB)	0/30 = **0%** (0%–12%)	0/40 = **0%** (0%–9%)	3/28 = **11%** (2%–28%)	1/19 = **5%** (0%–26%)	0/17 = **0%** (0%–20%)	0/39 = **0%** (0%–9%)
*Anaplasma* sp. (Omatjenne) (RLB)	1/30 = **3%** (0%–17%)	0/40 = **0%** (0%–9%)	11/28 = **39%** (22%–59%)	5/19 = **26%** (9%–51%)	0/17 = **0%** (0%–20%)	0/39 = **0%** (0%–9%)
*Ehrlichia ruminantium* (RLB)	0/30 = **0%** (0%–12%)	0/40 = **0%** (0%–9%)	0/28 = **0%** (0%–12%)	0/19 = **0%** (0%–18%)	0/17 = **0%** (0%–20%)	2/39 = **5%** (1%–17%)
*Theileria/Babesia* spp. (RLB)	15/30 = **50%** (31%–69%)	14/40 = **35%** (21%–52%)	28/28 = **100%** (88%–100%)	19/19 = **100%** (82%–100%)	14/17 = **82%** (57%–96%)	38/39 = **97%** (87%–100%)
*Babesia* spp. (1) (RLB)	1/30 = **3%** (0%–17%)	0/40 = **0%** (0%–9%)	0/28 = **0%** (0%–12%)	0/19 = **0%** (0%–18%)	8/17 = **47%** (23%–72%)	37/39 = **95%** (83%–99%)
*Babesia occultans* (RLB)	9/30 = **30%** (15%–49%)	0/40 = **0%** (0%–9%)	0/28 = **0%** (0%–12%)	0/19 = **0%** (0%–18%)	0/17 = **0%** (0%–20%)	0/39 = **0%** (0%–9%)
*Brucella* spp. (RBT and iELISA)	0/29 = **0%** (0%–12%)	0/40 = **0%** (0%–9%)	3/29 = **10%** (2%–27%)	0/21 = **0%** (0%–16%)	0/25 = **0%** (0%–14%)	0/35 = **0%** (0%–10%)
*Coxiella burnetii* (iELISA)	26/30 = **87%** (69%–96%)	16/40 = **40%** (25%–57%)	6/29 = **21%** (8%–40%)	14/21 = **67%** (43%–85%)	26/26 = **100%** (87%–100%)	18/39 = **46%** (30%–63%)
Foot-and-mouth disease virus (NSPCE)	1/29 = **3%***⁣*^*∗*^ (0%–18%)	0/40 = **0%** (0%–9%)	13/29 = **45%***⁣*^*∗*^ (26%–64%)	0/21 = **0%** (0%–16%)	Not tested	Not tested
*Theileria* spp. (RLB)	10/30 = **33%** (17%–53%)	0/40 = **0%** (0%–9%)	27/28 = **96%** (82%–100%)	19/19 = **100%** (82%–100%)	7/17 = **41%** (18%–67%)	33/39 = **85%** (69%–94%)
*Theileria bicornis* (RLB)	0/30 = **0%** (0%–12%)	0/40 = **0%** (0%–9%)	27/28 = **96%** (82%–100%)	19/19 = **100%** (82%–100%)	0/17 = **0%** (0%–20%)	1/39 = **3%** (0%–13%)
*Theileria buffeli* (RLB)	0/30 = **0%** (0%–12%)	0/40 = **0%** (0%–9%)	27/28 = **96%** (82%–100%)	19/19 = **100%** (82%–100%)	0/17 = **0%** (0%–20%)	1/39 = **3%** (0%–13%)
*Theileria equi* (RLB)	0/30 = **0%** (0%–12%)	0/40 = **0%** (0%–9%)	0/28 = **0%** (0%–12%)	0/19 = **0%** (0%–18%)	1/17 = **6%** (0%–29%)	1/39 = **3%** (0%–13%)
*Theileria* sp. (kudu) (RLB)	0/30 = **0%** (0%–12%)	0/40 = **0%** (0%–9%)	27/28 = **96%** (82%–100%)	0/19 = **0%** (0%–18%)	0/17 = **0%** (0%–20%)	0/39 = **0%** (0%–9%)
*Theileria* sp. (sable) (RLB)	11/30 = **37%** (20%–56%)	0/40 = **0%** (0%–9%)	25/28 = **89%** (72%–98%)	5/19 = **26%** (9%–51%)	0/17 = **0%** (0%–20%)	0/39 = **0%** (0%–9%)
*Theileria taurotragi* (RLB)	0/30 = **0%** (0%–12%)	0/40 = **0%** (0%–9%)	27/28 = **96%** (82%–100%)	0/19 = **0%** (0%–18%)	0/17 = **0%** (0%–20%)	0/39 = **0%** (0%–9%)

*Note:* Pathogens that have not been detected in any of the wildlife species are not included here. Bold indicates the prevalence for each pathogen and animal species which is the main outcome a reader is interested in.

Abbreviations: ELISA, enzyme-linked immunosorbent assay; iELISA, indirect ELISA; NSPCE, nonstructural protein competitive ELISA; RBT, rose bengal test; RLB, reverse line blot.

*⁣* ^*∗*^A subset of samples positive for foot-and-mouth disease virus (FMDV) based on nonstructural protein competitive ELISA (NSPCE) were tested for confirmation based on structural protein competitive ELISA (SPCE). All of these were negative by SPCE, including the wildebeest in Etosha and four kudus from Kruger National Park (KNP).

**Table 2:** Sample sizes, Bonferroni-corrected *p*-values, and *X*^2^ values of Pearson's chi-squared test with Monte Carlo simulation where prevalence has been used as outcome variable.

Pathogen (sample size)	Bonferroni-corrected *p*-values (*X*^2^ values)		
	Animal species	Sex	Sampling park
*Anaplasma bovis* (173)	1 (7.2)	1 (1)	1 (3.8)
*Anaplasma centrale* (173)	**<0.001 (75.1)**	1 (0.6)	0.152 (10.1)
*Anaplasma platys* (173)	1 (4.1)	1 (0)	1 (4.1)
*Anaplasma* sp. (Omatjenne) (173)	0.076 (16.5)	1 (0)	**<0.001 (14.9)**
*Babesia occultans* (173)	**<0.001 (45.3)**	1 (0.7)	0.38 (9.4)
*Babesia* spp. (1) (173)	**<0.001 (122.8)**	1 (1.5)	**<0.001 (23.7)**
*Ehrlichia ruminantium* (173)	1 (4.2)	1 (2.3)	1 (2)
*Ehrlichia/Anaplasma* spp. (173)	0.304 (14.6)	1 (0.4)	**<0.001 (22.4)**
*Theileria bicornis* (173)	**<0.001 (85.8)**	1 (0.1)	**<0.001 (65.3)**
*Theileria buffeli* (173)	**<0.001 (85.8)**	1 (0.1)	**<0.001 (65.3)**
*Theileria equi* (173)	1 (4.2)	1 (0)	1 (0)
*Theileria* sp. (kudu) (173)	**<0.001 (49.4)**	1 (0)	**<0.001 (32.4)**
*Theileria* sp. (sable) (173)	**<0.001 (26.7)**	1 (0)	0.076 (11.8)
*Theileria* spp. (173)	**<0.001 (33.8)**	1 (1.5)	**<0.001 (91.6)**
*Theileria taurotragi* (173)	**<0.001 (49.4)**	1 (0)	**<0.001 (32.4)**
*Theileria/Babesia* spp. (173)	**<0.001 (31.3)**	1 (1.9)	**<0.001 (54.9)**
*Brucella* spp. (179)	1 (4.9)	1 (0.3)	1 (3.4)
*Coxiella burnetii* (183)	**<0.001 (32.4)**	1 (1.7)	0.076 (14.9)
Foot-and-mouth disease virus (111)	1 (8.1)	1 (0.3)	**<0.001 (16.8)**

*Note:* Significant *p*-values are displayed in bold.

## Data Availability

Raw data are publicly available on Mendeley Data: https://data.mendeley.com/preview/ssf29pytwf?a=47d91a5e-2b3b-4764-8308-a3583af567bc. Questions about the data may be directed to the corresponding author.

## Supporting Information

Supporting Information 1 Table S1: Oligonucleotide probes fixed on the RLB membrane for the detection of *Anaplasma*, *Ehrlichia*, *Theileria*, and *Babesia* spp. DNA. References [53–56, 138–151] are here cited.

Supporting Information 2 Figure S1: Boxplots of (A) ELISA S/N percentages for foot and mouth disease virus (FMDV) and (B) ELISA S/P percentages for *Coxiella burnetii*. TH, threshold. Boxplot for *Brucella* spp. iELISA S/P percentages are not shown since some of the samples were tested in pools.
