# Supplementary material for: Detection of Tick-Borne Pathogen Coinfections and Coexposures to Foot-and-Mouth Disease, Brucellosis, and Q Fever in Selected Wildlife From Kruger National Park, South Africa, and Etosha National Park, Namibia
Source: Transbound Emerg Dis. 2024 Dec 12;2024:2417717. doi: 10.1155/tbed/2417717 (PMC12016786; doi:10.1155/tbed/2417717)
Supplement: Supporting Information 1 — Table S1: Oligonucleotide probes fixed on the RLB membrane for the detection of Anaplasma, Ehrlichia, Theileria, and Babesia spp. DNA. References [53–56, 138–151] are here cited. [file 2417717.f1.pdf]

Pathogen species	Probe sequence (5'-3')	Reference
Anaplasma bovis	GTA GCT TGC TAT GRG AAC A	(Bekker et al., 2002)
Anaplasma centrale	TCG AAC GGA CCA TAC GC	(Georges et al., 2001)
Anaplasma marginale	GAC CGT ATA CGC AGC TTG	(Bekker et al., 2002)
Anaplasma phagocytophilum	TTG CTA TAA AGA ATA ATT AGT GG	(Schouls et al., 1999)
Anaplasma platys	CGG ATT TTT GTC GTA GCT TGC TAT GAT	(Sirigireddy & Ganta, 2005)
Anaplasma sp. Omatjenne	CGG ATT TTT ATC ATA GCT TGC	(Bekker et al., 2002)
Babesia bicornis	TTG GTA AAT CGC CTT GGT C	(Zimmermann et al., 2021)
Babesia bigemina	GTA GTT GTA TTT CAG CCT CG	(Stoltsz et al., 2020)
Babesia bovis	CAG GTT TCG CCT GTA TAA TTG AG	(Gubbels et al., 1999)
Babesia caballi	GTG TTT ATC GCA GAC TTT TGT	(Butler et al., 2008)
Babesia canis	TGC GTT GAC CGT TTG AC	(Matjila et al., 2004)
Babesia divergens	ACT RAT GTC GAG ATT GCA C	(Matjila et al., 2008)
Babesia felis	TTA TGC GTT TTC CGA CTG GC	(Bosman et al., 2007)
Babesia genus-specific probe 1	ATT AGA GTG TTT CAA GCA GAC	(Matjila et al., 2008)
Babesia genus-specific probe 2	ACT AGA GTG TTT CAA ACA GGC	(Matjila et al., 2008)
Babesia gibsoni	CAT CCC TCT GGT TAA TTT G	(Bosman et al., 2007)
Babesia lengau	CTC CTG ATA GCA TTC	(Bosman et al., 2007)
Babesia leo	ATC TTG CTT GCA GCT T	(Bosman et al., 2007)
Babesia microti	GRC TTG GCA TCW TCT GGA	(Matjila et al., 2008)
Babesia occultans	CCT CTT TTG GCC CAT CTC GTC	(He et al., 2011)
Babesia rossi	CGG TTT GTT GCC TTT GTG	(Matjila et al., 2004)
Babesia sp. (sable)	GCT GCA TTG CCT TTT CTC C	(Oosthuizen et al., 2008)
Babesia vogeli	AGC GTG TTC GAG TTT GCC	(Matjila et al., 2004)
Ehrlichia canis	TCT GGC TAT AGG AAA TTG TTA	(Schouls et al., 1999)
Ehrlichia chaffeensis	ACC TTT TGG TTA TAA ATA ATT GTT	(Schouls et al., 1999)
Ehrlichia ruminantium	AGT ATC TGT TAG TGG CAG	(Bekker et al., 2002)
Anaplasma/Ehrlichia group-specific probe	GGG GGA AAG ATT TAT CGC TA	(Bekker et al., 2002)
Theileria annae	CCG AAC GTA ATT TTA TTG ATT TG	(Matjila et al., 2008)
Theileria bicornis	GCG TTG TGG CTT TTT TCT G	(Matjila et al., 2008)
Theileria buffeli	GGC TTA TTT CGG WTT GAT TTT	(Gubbels et al., 1999)
Theileria equi	TTC GTT GAC TGC GYT TGG	(Butler et al., 2008)
Theileria genus-specific probe	ATT AGA GTG CTC AAA GCA GGC	(Matjila et al., 2008)
Theileria lestoquardi	CTT GTG TCC CTC CGG G	(Nagore2004?)
Theileria mutans	CTT GCG TCT CCG AAT GTT	(Gubbels et al., 1999)
Theileria ovis	TTG CTT TTG CTC CTT TAC GAG	(Bekker et al., 2002)
Theileria parva	GGA CGG AGT TCG CTT TG	(Matjila et al., 2008)
Theileria separata	GGT CGT GGT TTT CCT CGT	(Schnittger et al., 2004)
Theileria sp. (buffalo)	CAG ACG GAG TTT ACT TTG T	(Oura et al., 2004)
Theileria sp. (kudu)	CTG CAT TGT TTC TTT CCT TTG	(Nijhof et al., 2005)
Theileria sp. (sable)	GCT GCA TTG CCT TTT CTC C	(Nijhof et al., 2005)
Theileria taurotragi	TCT TGG CAC GTG GCT TTT	(Gubbels et al., 1999)
Theileria velifera	CCT ATT CTC CTT TAC GAG T	(Gubbels et al., 1999)

Theileria/Babesia group-specific probe	TAA TGG TTA ATA GGA RCR GTT G	(Gubbels et al., 1999)
