# Supplementary figures and images for: Detection of Tick-Borne Pathogen Coinfections and Coexposures to Foot-and-Mouth Disease, Brucellosis, and Q Fever in Selected Wildlife From Kruger National Park, South Africa, and Etosha National Park, Namibia
Source: Transbound Emerg Dis. 2024 Dec 12;2024:2417717. doi: 10.1155/tbed/2417717 (PMC12016786; doi:10.1155/tbed/2417717)

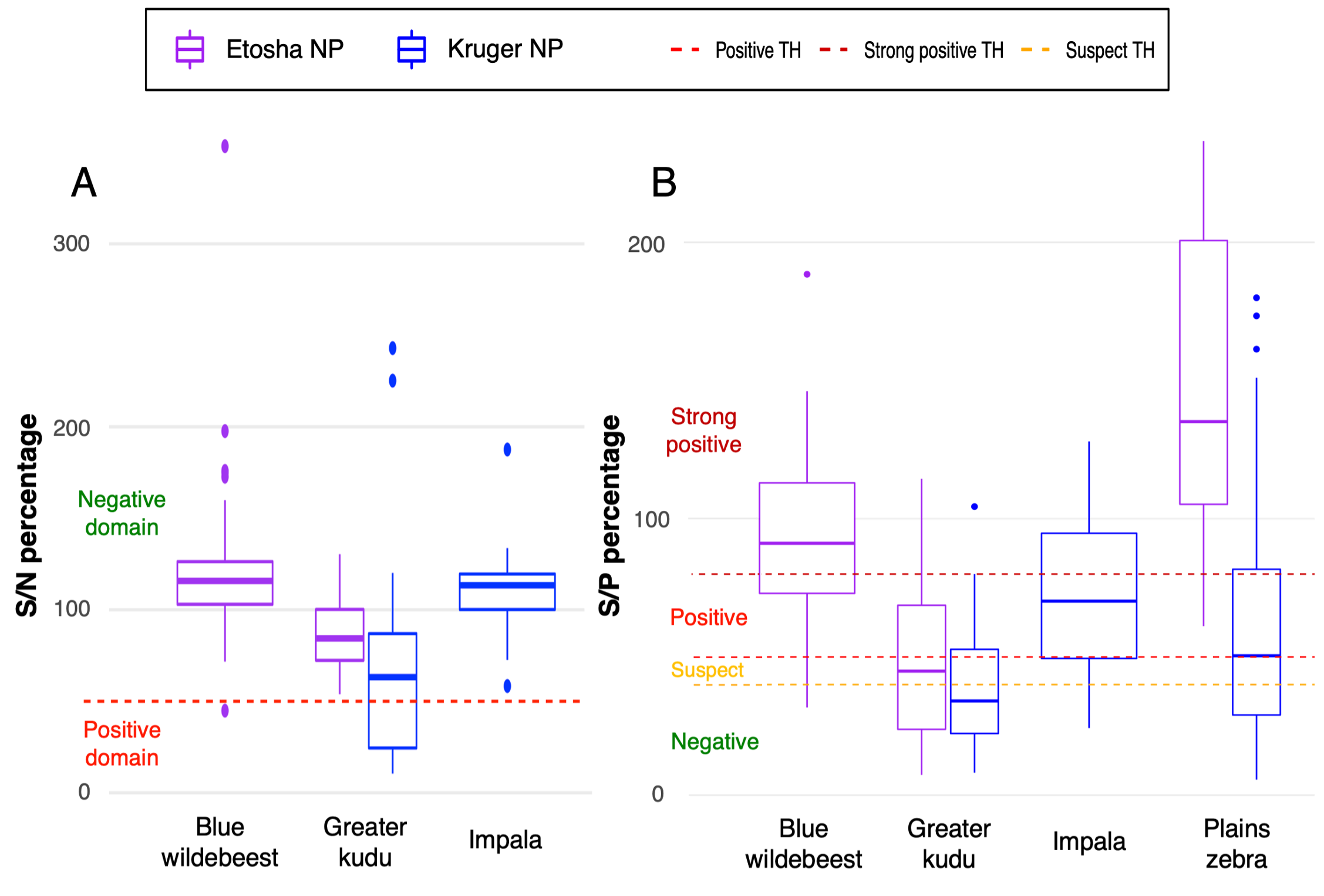

Supplement: Supporting Information 2 — Figure S1: Boxplots of (A) ELISA S/N percentages for foot and mouth disease virus (FMDV) and (B) ELISA S/P percentages for Coxiella burnetii. TH, threshold. Boxplot for Brucella spp. iELISA S/P percentages are not shown since some of the samples were tested in pools. [file 2417717.f2.png]
